# Latham’s Clinical Medicine

**Published:** 1847-10

**Authors:** 


### LATHAM'S CLINICAL MEDICINE.

We have barely had time to look into this volume, but one from the pen of this experienced physician and accomplished writer may safely be taken upon trust. It is a pleasure to read his works for their polished and vigorous style, betokening at once the scholar and the man of thought. They are models in their way, and it could be wished that such specimens were greatly multiplied. From our slight examination, we should say that it would not be easy for the student to find a more pleasant or instructive volume than the one before us.

